# Qualitative study of knowledge, perception, behavior and barriers associated with cardiovascular disease risk among overweight and obese Hispanic taxi drivers of South Bronx, NYC

**DOI:** 10.1186/s12889-020-08751-0

**Published:** 2020-05-14

**Authors:** Balavenkatesh Kanna, Aijan Ukudeyeva, Mohammad Faiz, Euripides Roques, Tina Washington, Leandro Ramirez, Masood A. Shariff, Maria Espejo

**Affiliations:** 1 Department of Internal Medicine, NYC Health + Hospitals/Lincoln, 234 East 149th Street, The Bronx, New York, 10451 USA; 2 ECRIP (Empire Clinical Research Investigator Program) Fellowship, NYC Health + Hospitals/Lincoln, 234 East 149th Street, The Bronx, New York, 10451 USA; 3 Center for Clinical and Community Research, NYC Health and Hospitals Corporation/Lincoln, 234 East 149th Street, The Bronx, New York, 10451 USA; 4 Department of Ambulatory Care, NYC Health + Hospitals/Lincoln, 234 East 149th Street, The Bronx, New York, 10451 USA

**Keywords:** Knowledge, Perceptions, Minority health, Taxi drivers, Cardiovascular disease

## Abstract

**Background:**

Taxi drivers are prone to developing cardiovascular disease (CVD) risk factors by adopting poor health behaviors due to their work environment. The population of Hispanic taxi drivers in inner city South Bronx, NYC, have not been studied. The goal of our qualitative study is to understand the perception, knowledge, behavior and barriers that influence CVD risk in overweight and obese inner-city Hispanic drivers.

**Methods:**

A cross-sectional qualitative study was conducted among community-based taxi drivers in South Bronx, NYC. Hispanic taxi drivers with body mass index of greater than 25 kg/m^2^ were screened and recruited for the study. Focus groups were organized to evaluate CVD and obesity risk factors through open-ended questions. The discussions were recorded, transcribed and analyzed using standard qualitative techniques. The Health Belief Model framework was applied to understand and evaluate likelihood of promoting health behaviors in this population based on the findings from the focus groups.

**Results:**

We conducted 3 focus groups (*N* = 25) and themes that emerged were evaluated. Through the Health Belief Model framework, Hispanic taxi driver participants reported demanding and stressful work shifts, barriers to better nutrition and good health, poor support systems, and low self-efficacy in overcoming barriers to improve their risk for CVD, due to lower perceived benefits and greater perceived barriers.

**Conclusions:**

Inner-city Hispanic NYC taxi drivers have several contributing factors and barriers leading to a poor CVD risk and high body weight profile. Understanding their knowledge, perception and barriers the drivers face in improving their CVD risk, underscores the importance of community outreach programs to develop a framework in empowering and improving the health of this population.

## Background

As per 2018 U.S. Bureau of Labor Statistics report, the number of taxi drivers nationwide is estimated to be 207,920, of which New York City (NYC) is home to 16,650 drivers [1]. Taxi driving is an occupation with a stressful lifestyle and work demands. Their overall income is low and requires them to work long hours, without health coverage, or paid vacations [2, 3].

Surveys conducted internationally and, in the US, have reported that taxi, truck and bus drivers are at higher risk of developing obesity, diabetes and hypertension [2, 4–7]. Studies on taxi drivers have shown higher prevalence of smoking, physical inactivity and unhealthy eating practices [8–15]. NYC taxi drivers have been found to have lower insurance coverage, faced discrimination, and higher prevalence of hyperglycemia and obesity [2, 15].

However, a detailed literature review shows that there are no studies focused on the health risks of Hispanic livery taxi drivers. The purpose of this study is to understand the perception, knowledge, health behaviors and barriers related to development of cardiovascular disease (CVD) risk factors in Hispanic taxi drivers in South Bronx, NYC. In partnership with NYC community-based organizations in the South Bronx, a Center for Health Evaluation, Education, Research and Engagement (CHEVERE) was launched to address the high prevalence of CVD risk factors in the predominantly minority population [16].

## Methods

A cross-sectional qualitative study design was developed to recruit Hispanic NYC livery taxi drivers and conduct focus groups. The subjects were screened and recruited from taxi bases with the help of the local Federation of Taxi Drivers in the borough of The Bronx. The recruitment methods were flyers, messages through taxi-base dispatch system radios, and referrals from livery taxi drivers and on-site active taxi-base recruitment. The research team developed a structured focus group protocol of open-ended interview questions related to cardiovascular disease. Study was reviewed and approved by the local Institutional Review Board (IRB) (IRB#15–016), and additionally each participant received a gift-card incentive of $35 at the conclusion of the focus group for their participation.

Participants were recruited based on the following inclusion criteria: age older than 18 years, body mass index (BMI) of greater than 25 kg/m^2^, working as livery taxi drivers in NYC for at least 6 months, and self-identified as Hispanic. Patients that participated in weight loss program or were on a weight loss trial, on medications for obesity, prior surgical history of weight loss surgery, history of current severe comorbidities leading to unintentional weight loss, diagnosis of severe disease or terminal illness, or driving outside of the five NYC Boroughs were excluded. These selection criteria’s were chosen to reduce confounders and facilitate a focused understanding of the perception, knowledge, behaviors, and barriers of at-risk taxi driver population.

Focus groups were planned to collect, transcribe, code, and analyze the themes through information gathering from a purposive sample of participants from the taxi driver organizations in the hospital catchment area of the South Bronx. A cross sectional random sampling technique was utilized. Multiple in person meetings were conducted at the taxi bases to provide study information to all taxi drivers in the base. Interested taxi drivers were randomly enrolled in the study. Four different researchers would analyze the recordings independently and inductively, adopting a thematic analysis approach to obtain different codes, categories, and themes until thematic saturation was achieved.

The participants were enrolled after being screened for eligibility into the study and informed consent obtained from each participant in their primary language by bilingual research staff trained in the study protocol and informed consent process. During recruitment, participants completed a baseline demographic, social, personal (tobacco use) and clinical history questionnaire, adapted from CDC’s BRFSS survey [17]. The BMI was calculated by measuring the weight and height of the participant (Weighing scale by Elite-My Weigh; Measuring tape by Telstar) during the screening process. Subjects selected a date to participate from a pre-scheduled set of dates and locations for attending the focus groups. The focus groups were held at the recruitment taxi bases or at the hospital site in a conference room with door(s) closed for privacy. The participants were given number tags that were used as a reference for each other and eventual coding. The groups were moderated by trained research team members in both English and Spanish according to the primary language spoken by participant. The research team that conducted focus groups were trained (Clinical Research Physicians) and coached to work on groups, utilize open-ended questions and avoid focus on personal risks of participants.

**Fig. 1:**
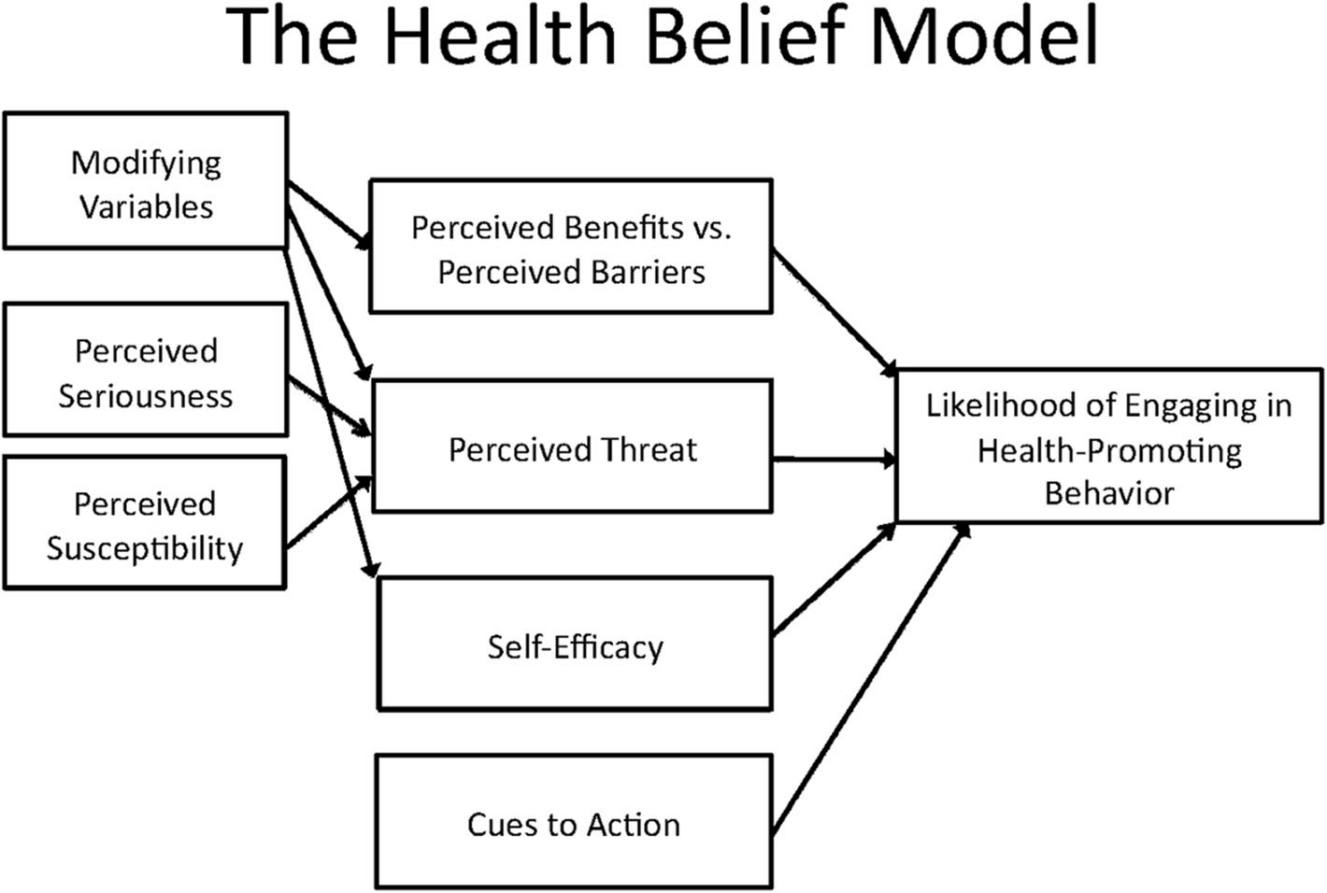
Application of Health Belief Model to focus group participants

The main categories of questions were organized based on perception, knowledge, behavior, and barriers. Participants were questioned on topics of etiology, risk perception, possible prevention and interventions related to CVD risks such as smoking, stress, obesity, diabetes, hypertension, and hyperlipidemia. Participants were queried with open ended questions to begin the discussion, and if participants did not elaborate on the initial question, a follow-up question was asked to clarify their response. Each focus groups lasted approximately for an hour and 30 minutes. Responses were recorded using audiotapes and transcribed verbatim. The conversations transcript was translated from Spanish to English by trained bilingual staff and analyzed using standard qualitative techniques with open code method. Four research investigators read the transcript separately and formulated concepts, which were then categorized and deduced into dominant themes. These themes were then compared and analyzed with a group consensus to ensure representative data was captured. Once recurring themes emerged and the saturation point was reached, study enrollment was stopped. In order to provide a conceptual framework and understand the complex interaction between knowledge, perception, health behaviors and the predisposition to CVD risk factors in our study population, the Health Belief Model (HBM) was employed. The HBM is one of the most utilized and easy to understand health models [18–20]. HBM is a widely recognized psycho-social model that has been used to understand, predict and modify health behavior. HBM helps to identify perception of risks of unhealthy behavior, barriers against healthy behavior, actions taken by patients to stay healthy, and commitment to their goals [21]. The HBM consist of six posits: 1) risk susceptibility, 2) risk severity, 3) benefits of action, 4) barriers to action, 5) self-efficacy, and 6) cues to action [22] (Fig. 1). According to the HBM, people’s beliefs about their risk and their perception of the benefits of taking action to avoid it, in turn influences their readiness to take those actions [20–25]. Study was conducted according to the COREQ guidelines for qualitative studies [26].

## Results

A total of 96 potential participants were approached. Of these, 34 did not meet the screening criteria (12 were new to the driving profession; 8 had BMI less than 25 kg/m^2^; 7 were not of Hispanic background; 5 worked only part-time as drivers; 2 were on a prescribed diet-plan to lose weight [with medications]) and the other 37 of the participants who were eligible, gave the following reasons to not participate: 12 were not interested; 7 did not have time; 5 not available to participate in focus groups session; 5 were no-shows to the scheduled focus group sessions; 4 stated they did not understand the purpose of the study; 2 stated the study was not a priority at this time; and 2 did not give a reason for refusal. Successive focus groups were held until thematic saturation was achieved and this point of saturation was reached after 3 focus group sessions including a total sample size of 25 subjects; there were 9 participants in Group-1; 8 in Group-2; and 8 in Group-3.

### Demographic data

All study subjects self-identified as belonging to Hispanic ethnicity, with a 92% male preponderance. Most taxi drivers participating in study were immigrants (96%), with a mean age of 53 years (ranged 21–69), and 92% preferred Spanish as their primary language. The participants also identified their race as White (20%), Black (4%), Other (52%), and remainder chose not to identify their race (24%). Mean BMI was 31.2 (25.3–38.7) kg/m^2^, where 56% were obese (BMI ≥30.0 kg/m^2^) and 44% were overweight (BMI 25.0 to 29.9 kg/m^2^). From this sample, 64% had high school education or higher, 50% had been diagnosed with hypertension and 27% reported a history of diabetes (Table 1).

**Table 1:** Baseline characteristics of the study participants (*N* = 25)

Age, years	
Mean ± SD	48.7 ± 13.3
Median (Range)	53 (21–69)
Gender	
Male	23 (92%)
Female	2 (8%)
Race	
White	5 (20%)
Black	1 (4%)
Other	13 (52%)
Refused	6 (24%)
Body Mass Index, kg/m2	
Overweight (25.0–29.9)	11 (44%)
Obesity (≥30.0)	14 (56%)
Marital Status	
Married	10 (40%)
Single	5 (20%)
Separated	6 (24%)
Widowed	3 (12%)
Unmarried Couple	1 (4%)
Education	
Grade 1–8	4 (16%)
Grade 9–11	4 (16%)
Grade 12/GED	13 (52%)
College 1–3 Yrs.	2 (8%)
College 4 Yrs. or More	2 (8%)
Annual Income	
< $29,000	17 (68%)
> $30,000	5 (20%)
Refused	3 (12%)
How Many Hours Do You Work Per Week?	
Mean ± SD	56.5 ± 15.8
Median (Range)	58 (35–88)
Coexisting Medical Condition^a^	
Hypertension	11 (44%)
Hypercholesterolaemia	11 (44%)
Diabetes	5 (20%)
Acute Myocardial Infarction	4 (16%)
Smoking History	3 (12%)

Data presented as n (%) unless otherwise noted. *GED* General Equivalency Diploma test, *SD* standard deviation. ^a^Each n (%) based on overall sample size

### Focus group study findings

The responses elicited from the focus groups were recorded, reviewed and deduced into the dominant themes based on concepts that emerged from the discussion (Table 2).

**Table 2:** Hispanic taxi driver: knowledge, perception, behavior, and barrier- questions and probes

Questions	Probes
Knowledge	
When you hear the word cardiac disease or heart disease what comes to mind? [Definition]	What about high blood pressure or hypertension, high blood sugar or diabetes, overweight or obesity?
What are some reasons that would put you at risk for heart disease, hypertension, or diabetes? [Etiology]	There are a number of conditions/behaviors (‘risk factors’) that may increase your chances of getting heart disease—such as physical inactivity, obesity and overweight, hypertension, diabetes, high cholesterol, and smoking—what do you think of these conditions and how they affect your chances of getting heart disease?
How do you think people get heart disease, diabetes, hypertension, obesity? [Cause]	What do patients think increases or decreases their risk for CVD? What are some things that cause CVD?
If a program could be developed and was designed especially for Hispanic livery taxi drivers to prevent CVD, what would it look like? [Intervention]	How would you teach people about CVD? How could we help teach others about CVD?
Perception	
How do you perceive CVD and its risks? [Self-perception]	Do you think that you are at risk for or have CVD? Why?
How does stress impact your health? [Stress]	Is it stressful working as a taxi driver? Tell me, how do you feel working? [Stress-occupational]
As a Hispanic livery taxi driver, what do you think of your risk/chances of getting heart disease? [Risk]	What are some reasons that would put you at risk for heart disease, hypertension, or diabetes? What do you think puts you at greater risk for getting heart disease?
Behavior	
How does stress impact your health? [Stress]	Is it stressful work in your job as a livery taxi driver? Tell me, how do you feel working? [Stress-occupational]
What can you do to prevent this risk? [Prevention]	What do you think about the role of lifestyle factors, including physical activity and diet, on modifying CVD risk? What prevents you from doing more exercise?
If a program could be developed and was designed especially for Hispanic livery taxi driver to prevent CVD, what would it look like? [Intervention]	How would you teach people about CVD? / How could we help teach others about CVD?
Barriers	
How do you feel about choosing your own decision on what is best for you based on what a physician offers you as treatment? [SDM]	Knowledge or information /Skills or Family help/support? What about computers? Face to face? [SDM-Sources]
When you are at work, what are some of the types of food you eat, and where? [Eating Habits]	How easy is it for you to eat while at work? What places do you choose to eat at? Why? Do you make your own food?

*CVD* cardiovascular disease, *SDM* shared decision making

Themes recorded during the focus group discussions include poor diet, sedentary lifestyle, comorbidities/risk factors, stress, and health are not being a priority, discipline, education, and intervention in that order of importance.

1. **Unhealthy Diet:** The theme “Poor diet” evolved from 151 related concepts that were described by participants. All 25 participants perceived their diet as unhealthy due to eating high fatty meals associated with the cultural food and selected restaurant chains that offered lower food prices and easy accessibility to parking. Drivers also reported that they did not have enough time to eat healthy foods based on their long working hours.

- Participants said: *“comemos muy tarde por que preferimos montar un pasajero…,”* stating that they preferred to pick up passengers and delay their meals.
- One other participant commented on the access to food in the neighborhood as follows: *Yo le voy a decir algo, yo he comido en todos los restaurantes del Bronx y en ninguno me siento satisfecho. Porque cuando yo pido arroz con habichuelas es aceite, sal y grasa. Ósea el arroz puede estar bien cocinado pero viene la carnes con la sal, grasa, salsa. La mezcla de alimentos es un veneno que nos estamos comiendo sin darnos cuenta. Eso es colesterol que nos estamos comiendo puro.”* (“I am going to tell you something, I have eaten in all the restaurants of the Bronx and in none I feel satisfied. Because when ask for rice with beans; it is oil, salt and fat, what I get. Because the rice may be well cooked but the meat comes with salt, fat, sauce. The food mixture is a poison that we are eating without realizing it. That is pure cholesterol that we are eating.”)

Another participant expressed concern about the quality of food:

- *“Se me olvido decir que además del pan y el café estamos acostumbrados al 3 golpes (huevo, salami, queso y mangú con mucha cebolla y aceite). Eso es algo que los taxistas comen todos los días en la mañana.”* (“I forgot to say that in addition to bread and coffee, we are used to three course breakfast [fried egg, fried salami, fried cheese and mangú with a lot of onion and oil]. That is something that taxi drivers eat every day in the morning.”)

All study participants considered poor diet as the most important factor in their increased risk for obesity, diabetes, and hypertension.

2. **Sedentary Lifestyle:** The theme “Sedentary Lifestyle” was derived from 147 similar concepts described by participants. They believe that physical inactivity is a leading risk factor for obesity, diabetes and CVD. Socio-economic and professional demands force them to drive an average of more than 10 h per day. They understand the importance of daily exercise but admitted that at the end of the day they were too fatigued to exercise or the time spent on exercising could rather be utilized to work more hours thus increasing their income.

- Participants said, *“Ahora mismo estamos en un estado muy vulnerable porque pasamos el día entero sentado y no nos preocupamos por la salud. Nos preocupamos mas por otras cosas. Llegamos a la casa nos bañamos y nos acostamos a ver televisión y no pensamos en nosotros mismos. Solo pensamos en el trabajo y llegar a la casa a descansar. Creo que estamos tomando bastante riesgo con eso.”* (“Right now, we are living vulnerable times because we spent the entire day sitting around and we do not worry about our health. We worry more for other things. When we arrive home, wash up, and go to bed to watch TV without “thinking” of our health. I think we are taking a lot of risk with our health.”)

3. **Greater risk for comorbidity:** The theme “Comorbidities” developed from 143 concepts grouped together. Taxi drivers were able to correlate that obesity can lead to future complications affecting many vital organs, such as the kidneys, the heart and vasculature. Furthermore, they perceived obesity as important risk factor for high blood sugar and cholesterol levels. They saw an association between the condition of their overall health and work, however, the financial wellbeing of their families rather than their health, took priority. They also understand that family history of obesity and CVD increases their risk of obesity, diabetes and cardiovascular risks.

- In participants’ own words, *“Me viene a la mente el colesterol, las arterias, la presión, el corazón. Todo lo que tiene que ver con el acondicionamiento de los problemas cardiovasculares. Entiendo que la diabetes, obesidad, presión alta es una bomba de tiempo. Si no son tratadas, duramos poco.”* (“Cholesterol, arteries, blood pressure, and heart health come to mind. Everything we do has to do with the condition of cardiovascular problems. I understand that diabetes, obesity, high blood pressure is a ticking time bomb. If they are not treated, our lives will be shortened.”)

4. **Work Stress:** The theme “Stress” was derived from 141 concepts. Taxi-drivers perceive their profession with lack of organization and high stress levels as one of the leading risk factors contributing to obesity, diabetes and cardiovascular disease. They also attribute a combination of stressful lifestyle, poor diet, lack of exercise, consumption of alcohol and cigarettes as determining factors in developing negative health outcomes.

One participant said: *Tenemos el paquete complete…* (we have the entire package…).

- In their leisure time they shared that they indulged in alcohol, feasting and sedentary habits, and stated: *…Y como pasamos sentados todo el día con la presión del pasajero, del trafico, de la policía, de los inspectores de taxis. Cuando llegamos a la casa, destapamos una botella de romo o nos ponemos a ver television …Especialmente los Dominicanos que nos gusta darnos “petacazo.”* (“...And as we spend all day sitting with the pressure of the passenger, traffic, police, and taxi inspectors. When we arrive at the house, we uncover a bottle of Rum or we start watching television ... Especially the Dominicans who like to binge drink alcohol.”)

5. **Health not a priority:** The theme “Health is not a priority” was derived from 120 concepts based on the taxi drivers’ responses. Taxi drivers prioritize their work while their health takes a back seat. They work long shifts in response to the pressures of the financial responsibilities to their family, they admit to a lack of intention to change their behavior and consider themselves as “hard headed.” Financial responsibility and commitment to their family is more important than themselves and their health. Drivers changed their behavior only when serious health conditions related to obesity, diabetes and hypertension develop that require professional medical attention.

- They said, *“La necesidad de satisfacer los distintos gastos que tiene uno en casa y alrededor de uno. Creo que no nos hace preocuparnos mas por la salud…. Nosotros los taxistas pensamos que si nos paramos 1 hora o pal de minutos como es empezar de nuevo y mejor prefiere quedarse sentado en el carro a ver que aparece hasta que complete las horas que uno trabaja en el día.*” (“The need to meet the different expenses that one has at home and around one. I think it doesn’t make us worry more about health… We taxi drivers think that if we stop for an hour or even few minutes it is a start over; so, we better prefer to sit in the car to see what comes our way until we complete the work hours in the day.”)

6. **Lack of personal discipline:** The theme “Discipline” evolved from 80 concepts derived from the driver’s transcripts. Taxi drivers are aware of their lack of organizational skills in general, especially when it comes to the balance between work and a healthy lifestyle. Taxi drivers recognize that not being disciplined about healthy behaviors contributes to the development of their obesity and chronic health conditions. Drivers admit that they don’t have a fixed schedule, with no direct supervision, and cannot find the time to go to the doctor or change their behavior. Lack of time is an important barrier in pursuing preventative care.

- Participants stated, *“El 90% de los taxistas, son una bomba de tiempo. Porque? Porque comemos muchisima porqueria, comida chatarra. La mayoría de nosotros no comemos en la casa, es en la calle. A veces vamos a restaurantes de comida rápida a comer y eso es fatal principalmente para lo que tienen, que son diabéticos, los que sufrimos de problemas cardiacos. Por esa razon ademas de ser sedentario tambien lo que entra por aqui (la boca) nos hace mucho dano*.” (“The 90% of taxi drivers are a ticking time bomb. Why? Because we eat a lot of junk food. Most of us do not eat at home, it is on the street. Sometimes we go to fast food restaurants to eat and that is fatal mainly for those who are diabetic, and those who suffer from heart problems. For that reason, and in addition to being sedentary also, what comes through in here (the mouth) causes a lot of damage.”)

7. **Health Education:** The theme “Education” was derived from 79 concepts noted from the focus group discussion. Taxi drivers know that their lack of health education is affecting them. They expressed poor understanding about the severity of the disease process that makes it difficult for them to take proactive measures to prevent disease progression. They are interested in the development of programs that will educate them about obesity, diabetes and CVD prevention and desire to attend programs that can educate them about prevention with strong emphasis on healthy eating. They understand that this would increase their ability to change their unhealthy behavior.

- Participants’ comments include: *“Yo como lo que aparezca. Nosotros no nos ilustramos con relación a que es saludable y no. Pero en verdad, nosotros mucha veces no sabemos que es comer saludable. Tu no sabes si es saludable para tu cuerpo.”* (“I eat what I find. We are not educated in relation to what is healthy and not. We often don’t know what it is to eat healthy. You do not know if it is healthy for your body.”)

8. **Health interventions:** The last major theme “Intervention” was derived out of 71 concepts. When asked about possible interventions that might help them towards healthy behaviors, taxi drivers think that the use of technology as a means of education is very effective. They understand the most direct route to reach them is by cell phone, email and social media such as Facebook, Inc. They also feel that it would be good to use this type of communication to not only to inform them about health issues, but to also educate them directly.

- Participants stated, *“Yo creo que si la cuidad con el estado hiciera un tipo de restaurantes que el taxista pudiera ir a comer saluda como un buffet. Que uno pueda parquearse, sin estrés, comer tranquilo, comer saludable, no comer arroz, habichuelas y carnes. Sino variar. Que no sea lo mismo. Yo creo que es una buena opción y la cuidad se beneficia también porque nosotros vamos a consumir.”* (“I think that if the city/state governments would create restaurants that sells healthy food buffet, taxi drivers could afford it and eat healthy. Some place that we could park without stress, eat peacefully, but no rice, beans and meat, and something different. I think this is a good idea and the city would benefit too because we are going to buy and consume.”)
- *Estoy de acuerdo con el otro taxista…Los restaurantes y la comida rápida son lo primero que enferma a la gente en esta cuidad.* (“I agree with the other driver [the above comment] … The restaurant and fast food chains are the primary reason people get [CVD] in the city.”)

## Discussion

This is the first qualitative study to evaluate cardiovascular disease risk among overweight and obese Hispanic taxi drivers in NYC. The study findings, utilizing HBM conceptual framework, shows us that Hispanic taxi drivers perceive several barriers to healthy lifestyle, such as a lack of education and knowledge about healthy food choices, places where they can buy healthy affordable snacks, information about physical activities, stress management skills, poor support systems, and organizational skills.

All taxi driver participants of our study self-reported their ethnicity as Hispanic with mixed racial profile, and all but two participants were males. This finding concurs with data in literature, which shows that an overwhelming number of taxi drivers in NYC are minority men, with 84% being immigrants and 98% identify as male [2].

Even though the median household income in the Bronx borough is $35,302 [27], 68% of our group of taxi driver participants reported income below this income range. Majority of the Hispanic taxi drivers had lower than high school education, and worked long hours every week to make ends meet. Many taxi drivers reported pre-existing cardiovascular co-morbidities such as hypertension and diabetes in addition to overweight or obesity. Our finding corroborates other studies that have reported that taxi, truck and bus drivers are at a higher risk of developing diabetes and hypertension [2, 4–7]. Interestingly, smoking rate was much lower in our study participants than published rates, the specific reason being unclear, but could be related to the impact of public health messaging in this community.

The results of our focus group sessions demonstrate that the perceptions, knowledge, beliefs and barriers noted among NYC Hispanic Taxi drivers in accordance to the following six constructs or posits of the HBM may be associated with a lower likelihood of adopting health promoting behaviors.

- In terms of first HBM construct namely, increased risk susceptibility, many drivers perceive their susceptibility to cardiovascular disease and obesity risks. The participants contribute this to their sedentary occupation, consumption of unhealthy diet and finally financial constraints that limit their options to buy healthy accessible, affordable and culturally familiar cuisines.
- Their reported behavior demonstrates limited perception of risk severity, the second HBM posit, as they admit that physician visitation and accepting their recommendations became a priority only during times of sickness or illness.
- The benefits of healthy behaviors were not fully understood by taxi drivers, thus illustrating the third posit of the HBM, namely low understanding of the perceived benefits. We believe that healthy behavior was a secondary concern for the drivers because of work fatigue, family obligations and responsibilities and lack of time, financial incentives and higher education (84% of drivers had only secondary education) as mentioned during the focus group settings.
- The fourth HBM posit encompasses an understanding of the potential negative aspects of a particular health action and in our study we learned that even though most participants understood the importance of implanting healthier behaviors (e.g. physical activity, healthy food consumption), however they still failed to engage in physical activity or seek out healthy food options, citing financial constraints and busy work schedule on their own.
- The fifth construct of HBM is cues for action, which are environmental triggers that act on individual’s perception that lead to change in behavior. In the focus groups, we learned that drivers were likely to visit physicians or change their unhealthy habits when one of their family member, friend or other taxi driver had suffered some form of “physical pain or illness.” Other positive cues to action was their willingness to receiving educational materials provided by either their physicians or community health advocates using electronic media, cell phones or mass emails for disease prevention.
- The sixth HBM posit is related to self-efficacy, which was lacking in our focus group participants. For example, taxi drivers expressed lack of confidence when asked if their behavior can be changed, they cited poor discipline, lack of organizational, and time management skills as few reasons for this drawback. However, they were receptive to receiving information about time management, healthy snacks alternatives, availability of affordable, and healthy food options, and learning more about different physical activities and exercise regiments.

Thus, through the application of the HBM posits, our study shows that NYC Hispanic taxi drivers, even though perceived their risk susceptibility, lack self-efficacy, lack perceiving benefits of action, lack an understanding of cues to action, and understanding the risk of disease severity placing them at greater risk for factors that lead to CVD. They acknowledge that poor diet, stressful work environment, lack of organizational skills, increased competition from ridesharing services [28], and working extended hours are some of the barriers that contribute to their susceptibility of CVD. Furthermore, participants related that in their leisure time, consumption of alcohol, smoking cigarettes, sedentary lifestyle rather than engaging in physical activity were other factors that made them prone to being overweight and obese as elicited during the focus groups.

Our findings utilizing the HBM conceptual framework shows where opportunity for intervention lies and how we can intervene and modify the health behavior of the at-risk NYC Hispanic taxi driver population. Any future interventions will have to encompass strategies to address the above listed barriers.

Our study’s limitations include a small sample size, but we reached a saturation point within this sample. Excluded non-participants could have been healthier than participants and choose not to participate because they saw little benefit. Alternatively, non-participants could have been less healthy and felt embarrassed to participate, leading to an underestimation of CVD risk. In addition, the majority of participants in the current study were 40 years or older; responses among younger drivers may have been different. The list of themes presented is not an exhaustive list and further research is required on other themes that could potentially impact health behavior and outcomes. Differences in occupational demands reported by yellow taxi and livery car service drivers suggests that focus groups’ specific to each group could yield different views.

## Conclusion

By obtaining a better understanding of the link between behaviors of this population, perception, knowledge, and barriers utilizing a health behavior model framework, our study highlights the predisposition for CVD risk in overweight and obese Hispanic NYC taxi drivers. Taxi drivers perceive risk factors and possess knowledge about the consequences of those health risks. Utilizing the HBM constructs, taxi drivers are found to have low self-efficacy, lack of perceived benefits of the value of engaging in a health-promoting behavior to decrease risk or have limited understanding of cues to action and greater number of perceived barriers. Barriers to improving their health include unhealthy lifestyle due to stressful and high demand work environment, for which they require support and programs targeted to improve this risk for CVD.

## Data Availability

All study data-collection and analysis has been presented in the manuscript. Surveys and Open-Ended questions can be provided upon request.

## Abbreviations

BMI: Body mass index
BRFSS: Behavioral Risk Factor Surveillance System
CDC: Centers for Disease Control and Prevention
CHEVERE: Center for Health Evaluation, Education, Research and Engagement
COREQ: Consolidated criteria for reporting qualitative research
CVD: Cardiovascular disease
HBM: Health Belief Model
IRB: Institutional Review Board
NYC: New York City
